# Impact of Sulforaphane on Breast Cancer Progression and Radiation Therapy Outcomes: A Systematic Review

**DOI:** 10.7759/cureus.78060

**Published:** 2025-01-27

**Authors:** Nada Alhazmi, Ahmad Subahi

**Affiliations:** 1 Basic Sciences, College of Science and Health Professions, King Saud Bin Abdulaziz University for Health Sciences, Jeddah, SAU; 2 Research Office, King Abdullah International Medical Research Center, Jeddah, SAU

**Keywords:** apoptosis, breast cancer biology, breast cancer progression, cancer stem cells, metastasis, radiation therapy, reactive oxygen species, sulforaphane

## Abstract

Breast cancer is a debilitating chronic condition that affects millions and causes the death of hundreds of thousands of people annually worldwide. Sulforaphane (SFN) has the potential to prevent and enhance the management of breast cancer and alleviate its poor outcomes. As most of the available evidence on SFN’s impact and safety in breast cancer management is from cell culture studies and animal models, there is a need to consolidate this evidence to determine whether there is a need to conduct empirical human studies. This systematic review summarizes and analyzes evidence of SFN’s impact on breast cancer progression and radiation therapy outcomes in terms of efficacy and safety. A systematic literature search was performed on PubMed, Cumulated Index in Nursing and Allied Health Literature, ProQuest Central, and ScienceDirect databases to find sources of evidence. The selected sources were published between 2018 and 2024. The inclusion and exclusion criteria included studies published in the last eight years, availability in full-text, published in English, and covering concepts relevant to the topic. The selected sources were summarized through literature review tables and the evidence was synthesized to identify themes relevant to the impact of SFN in breast cancer progression and radiation therapy outcomes. In total, 20 sources of evidence, including three randomized controlled trials, five in vivo animal models, and 12 cell culture studies, were selected for review and analysis. The sources were broadly classified into two themes, namely, the role of SFN in breast cancer biology and the safety of SFN in radiation therapy. The evidence confirmed that SFN targets breast cancer biology by modulating reactive oxygen species and reducing the proliferation of tumors, inducing apoptosis, and inhibiting metastasis. SFN is an effective and safer treatment as it reduces damage to and protects normal cells, causes a synergistic effect rather than interacting with other breast cancer cells, and is effective in concentrations ranging from 5 µM to 200 µM. Cell culture studies and animal models have proven that SFN is highly effective in breast cancer prevention and treatment either as a single intervention or in combination with radiotherapy and chemotherapy.

## Introduction and background

Breast cancer is a debilitating condition that affects a large number of people within the community. Breast cancer occurs when there is an abnormal or uncontrolled cell growth that results in the formation of a tumor in the breast tissue [1]. The condition is classified into luminal A, luminal B, human epidermal growth factor receptor 2 (HER2)-enriched, and basal-like main molecular subtypes differentiated by fewer mutations and changes in chromosomal copy numbers, more mutations and changes in chromosomal copy numbers, high levels of HER2 protein, and greater aggressive accompanied by poor prognosis, respectively [2]. Regarding histopathologic subtypes, breast cancer is classified as triple-negative breast cancer (TNBC) or ductal, lobular, papillary, medullary, mucinous, tubular, comedo, or inflammatory carcinoma depending on whether it originates from the milk ducts, originates from lobules, has finger-like projections of cancer cells, has a distinct appearance with better prognosis, non-invasive and early stage, and rapidly spreading through the lymphatic system.

Breast cancer is currently the most commonly diagnosed type of cancer among females in the United States [1]. Data from the Global Cancer Observatory shows that breast cancer accounts for 2.3 million or 11.6% of the 19.96 million new cancer cases reported worldwide each year. The data further show that 665,684 new cancer deaths are reported each year. Although the mortality rate of breast cancer is almost similar in the Middle East (Western Asia) compared to regions such as Northern America, Australia, and New Zealand at 15.1 per 100,000, its incidence is relatively lower in the Middle East (Western Asia) at 45.4 per 100,000 or less than half of the above comparable regions. Luckily, the mortality rate associated with breast cancer has dropped consistently in recent years at a time when its incidence has shown a rising trajectory. Statistics from developed economies show that breast cancer accounts for almost a third of all new cancer cases diagnosed among women each year and is a key cause of economic burden among patients and the healthcare system [2]. The key to successfully treating patients with breast cancer lies in early diagnosis and the use of safe and effective treatment modalities [2].

Radiation therapy is an effective treatment approach for patients diagnosed with breast cancer. Radiation therapy is indicated after lumpectomy to alleviate the risk of breast cancer recurrence or manage metastasis after mastectomy [3]. This intervention is indicated in 70% of patients receiving breast cancer treatment [3]. Unfortunately, prolonged exposure to this intervention is known to expose patients to health risks and may not effectively treat all patients [4]. As such, different adjunctive therapy options have been explored to enhance patient outcomes during breast cancer treatment. Sulforaphane (SFN) is among the adjunctive interventions that have shown the potential to enhance breast cancer treatment and reinforce the effects of radiation therapy [5].

SFN is a naturally occurring isothiocyanate present in cruciferous vegetables such as kale, broccoli, and sprouts [6]. The intervention is used as a dietary component whose natural bioactive botanic components have shown promising results in breast cancer management [3,6]. Empirical evidence suggests that SFN can reduce tumor sphere formation within cancer stem-like cells (CSCs) and malignant cell proliferation when used individually and leads to a synergistic effect on the suppression of breast cancer when co-administered with radiation therapy at optimal concentrations [3]. SFN also enhances the expression of cyclin B1 proteins, inducing G2/M-cell cycle arrest and reducing the proliferation of cancerous cells. The compound also activates poly (ADP-ribose) polymerase 1 and caspase family proteins present in CSC lines, thereby inducing apoptosis and inhibiting tubulin polymerization in breast cancer cells [6]. In essence, SFN inhibits the growth of CSCs and induces their death. As such, SFN may be a beneficial chemotherapeutic intervention for breast cancer treatment.

Many existing studies have based their evidence on cell culture and animal models that lack adequate validity to use as sources of evidence-based practice (EBP) [7-10]. However, the validity of this evidence could be enhanced by systematically consolidating the evidence from single empirical studies. Although the effectiveness of SFN as an antimicrobial agent, an adjunctive preventive agent for various types of cancer, and an anti-inflammatory agent has been proven in the last two decades, research on the effectiveness of the compound as a treatment for various forms of cancer commenced just a few years ago [6]. The scarcity of evidence on the effectiveness of SFN calls for a review of the few existing studies to determine the need for, feasibility of, and safety of clinical trials on the compound. A systematic review on the topic could help identify consistencies or inconsistencies in single studies exploring SFN’s role in breast cancer cell biology and the beneficial effects of combining the intervention with radiation therapy. This systematic review aims to consolidate preclinical evidence and mechanistic studies on the role of SFN in modulating breast cancer progression, therapy resistance, and treatment outcomes, with a focus on the interplay between SFN and radiation therapy.

## Review

Methodology

This study followed the Preferred Reporting Items for Systematic Reviews and Meta-Analysis (PRISMA) protocol for systematic reviews. This study design was selected as it allowed synthesizing evidence from multiple sources to present a summary that clarifies the effectiveness of SFN in breast cancer management. A systematic search was performed between November 7th and 10th, 2024, using search terms to necessitate the identification and selection of sources relevant to the topic. These search terms included “sulforaphane,” “breast cancer,” breast tumor,” “metastasis,” “apoptosis,” “reactive oxygen species,” and “radiation therapy.” These terms were combined using the Boolean operators AND/OR where allowed by the databases used for this research. The PRISMA chart was used to document the search process and facilitate the selection of high-quality evidence.

Inclusion and Exclusion Criteria

Inclusion and exclusion criteria were used to narrow down the search results. The use of strict inclusion and exclusion criteria prepared before the search helped avoid bias in the selection of sources. Interestingly, each of the selected databases necessitated the application of specific criteria to filter the most relevant sources. First, all sources had to be peer-reviewed journal articles as the rigorous process that they are subjected to before publication ensures that they present valid and reliable evidence. All sources had to be published in the English language to reduce the risk of loss/distortion of meaning when translating the information. All sources had to be published within the last eight years (2017-2024) to ensure currency and relevance of evidence. All sources had to be available in full text to necessitate access to all relevant information. Databases such as PubMed only published abstracts but provided links to full-text articles. In such cases, sources were considered as long as the full text was available and accessible. Sources were excluded if their publication language was not English and if they were older than eight years, from non-peer-reviewed journals, irrelevant to the topic, and unavailable as full-text articles.

Search Strategy and Study Selection

The PubMed, Cumulated Index in Nursing and Allied Health Literature (CINAHL), ScienceDirect, and ProQuest Central digital databases were used to search articles for the review relevant to the selected topic. These databases were selected as they publish peer-reviewed articles from numerous peer-reviewed journals relevant to biomedical research. The search was performed using the search engine available in each of these databases while built-in filters were used to enforce the inclusion and exclusion criteria. The filters included sources published in the last eight years, availability in full text, and publication in the English language.

Search results

**Figure 1:**
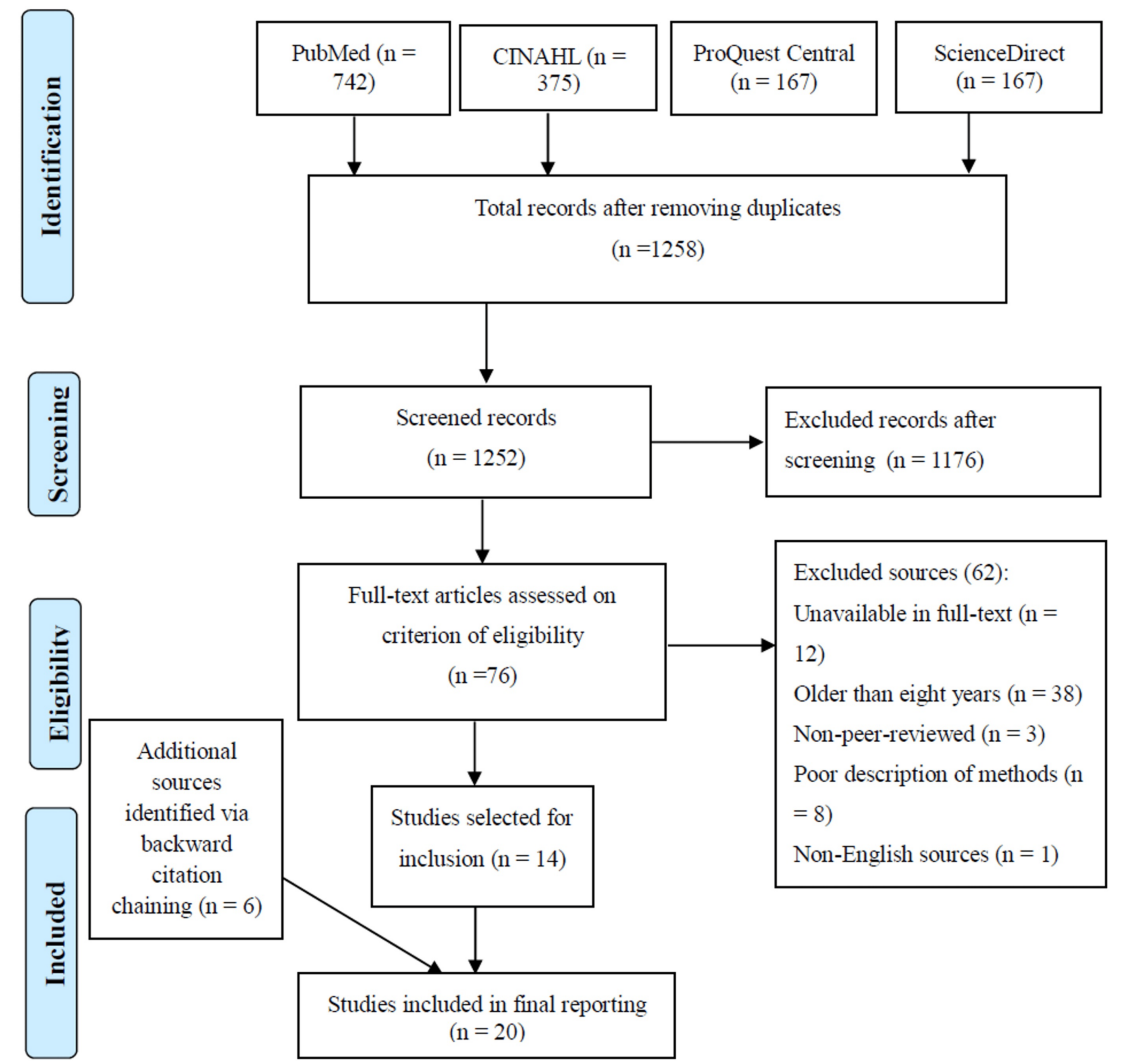
Preferred Reporting Items for Systematic Reviews and Meta-Analysis flow diagram. Original image created by the authors.

After the search strategy and selection were applied, 20 of the most relevant sources were selected for inclusion. Figure 1 is an illustration of the PRISMA flow diagram that was used to record the search process. The Johns Hopkins Nursing Evidence-Based Practice tools were used to appraise the sources for quality and level of evidence.

The search yielded 1,451 sources from four databases. These results included 742, 375, 167, and 167 sources from PubMed, CINAHL, ProQuest Central, and ScienceDirect databases, respectively. However, 199 sources were eliminated due to duplication. The remaining 1,252 sources were screened, of which 1,176 were eliminated as they were deemed irrelevant to the topic. The remaining 72 sources were further evaluated for evaluation. Of these, 12, 38, three, eight, and one of the sources were eliminated due to unavailability in full-text, being older than eight years, being non-peer-reviewed, and having been published in a non-English language, respectively. Finally, the search identified 14 articles. However, this number was viewed as too low for the systematic review. As such, six additional sources were identified through backward citation chaining to bring the final search results to 20 high-quality, peer-reviewed, and current sources of evidence. These sources included three randomized controlled trials (RCTs), five in vivo animal models, and 12 cell culture studies. Table 1 and Table 2 summarize these studies with a focus on methodologies, outcomes measured, findings, and conclusions.

**Table 1:** Summary of evidence on the role of sulforaphane in breast cancer biology. SFN: sulforaphane; CSC: cancer stem cell; NF-κB: nuclear factor kappa B; HER2: human epidermal growth factor receptor 2; TNBC: triple-negative breast cancer; ALDH+: aldehyde dehydrogenase-positive; LSD1: lysine-specific demethylase 1; HDAC5: histone deacetylase 5; ALDH1A1: aldehyde dehydrogenase 1A1; ROS: reactive oxygen species; Nrf2: nuclear erythroid 2-related factor 2; TGF-β1: transforming growth factor beta 1

Author (year), country	Histopathologic breast cancer type	Article title	Methodology, sample size	Patient outcomes	Findings/Conclusions	Implications
Burnett et al. (2017) [1], United States	Breast anaplastic carcinoma	Sulforaphane enhances the anticancer activity of taxanes against triple negative breast cancer by killing cancer stem cells	In vitro cell culture, N = 2	Increased cell death; reduced proliferation; breast cancer cell metastasis	SFN preferentially eliminates breast CSCs by suppressing or downregulating NF-κB p65 subunit translocation, downstream transcriptional activity, and p52 proteins. SFN reverses ALDH+ cell enrichment induced by taxane and consequently reduces the volume of resultant primary and secondary mammospheres formed. Combining SFN and taxanes increases the suppression of secondary tumor formation. The ddition of SFN to conventional breast cancer treatments can prevent the expansion of and facilitate the elimination of breast CSCs	Potential for therapy; implications for treatment; reduced metastasis risk
Cao et al. (2019) [2], United States	TNBC	HDAC5-LSD1 axis regulates antineoplastic effect of natural HDAC inhibitor sulforaphane in human breast cancer cells	In vitro cell culture, N = 1,208	Reduced proliferation; reduced metastasis risk; activation of key tumor-suppressive signaling pathways	SFN facilitates lysine-specific demethylase 1 (LSD1) ubiquitination and degradation in an HDAC5-dependent manner which inhibits breast cancer progression. Combining SFN with a potent LSD1 inhibitor results in synergistic growth inhibition in tumors with no impact on normal breast epithelial cells. Combining SFN and LSD1 inhibitors accelerates the inhibitory effect on MDA-MB-231 xenograft tumor growth. SFN enhances HDAC5-LSD1 axis inhibition and blocks breast cancer growth	Potential for therapy; implications for treatment; reduced metastasis risk
Castro et al. (2020) [2], United States	HER2-enriched breast cancer	Sulforaphane suppresses the growth of triple-negative breast cancer stem-like cells in vitro and in vivo	In vivo animal model, N = 16	Increased cell death; reduced proliferation	SFN inhibits cell proliferation and mammosphere formation of CSCs in TNBC. SFN significantly decreases the expression of cancer-specific CR1 and CRIPTO-3/TDGF1P3 genes. SFN suppresses the expression of stem cell markers Nanog, ALDH1A1, Wnt3, and Notch4. SFN may control CSC malignant proliferation in TNBC via CRIPTO-mediated pathway by inhibiting CRIPTO/Alk4 protein complex formation and suppressing its expression and is thus suitable for the chemoprevention of TNBC	Potential for therapy; implications for treatment; reduced metastasis risk
Gong et al. (2020) [4], China	Ovarian carcinoma	Sulforaphane enhances the cisplatin sensitivity through regulating DNA repair and accumulation of intracellular cisplatin in ovarian cancer cells	In vitro cell culture, N = 12	Increased cell death by causing DNA damage to cancerous cells; reduced tumor proliferation	SFN reverses carcinoma cell resistance to cisplatin that may be induced by DNA damage. Additionally, it increases the accumulation of intracellular cisplatin. SFN treatment increases miR-30a-3p expression in cisplatin-resistant cells. SFN enhances A2780/CP70 and IGROV1-R10 cells’ sensitivity to cisplatin treatment. SFN is a key regulator for DNA repair and cisplatin transportation during cancer treatment	Potential for therapy; implications for treatment; reduced metastasis risk
Keshandehghan et al. (2020) [5], Iran	HER2-enriched breast cancer	Co-treatment with sulforaphane and nano-metformin molecules accelerates apoptosis in HER2+ breast cancer cells by inhibiting key molecules	In vitro cell culture, N = 96	Increased cell death; reduced proliferation	There was a direct correlation between SFN’s effect on cell death and HER2 levels, with a higher number of cell deaths being reported at higher concentrations of SFN. Cell co-staining indicated that the percentage of apoptosis correlates that were destroyed following different treatments was high, with greater outcomes being reported in patients treated with SFN compared to metformin. Combining SFN with metformin synergized particular molecules or cell signaling pathways and showed their cooperative effects on cancer cells. SFN reduces cancer cell viability which can be enhanced by adding metformin by targeting common molecules/pathways downstream of HER2 and may be key for CSC signaling	Potential for therapy; implications for treatment; reduced metastasis risk
Kim et al. (2017) [6], South Korea	TNBC	Sulforaphane sensitizes human breast cancer cells to paclitaxel-induced apoptosis by downregulating the NF-κB signaling pathway	In vitro cell culture, N = 24	Increased cell death; reduced tumor proliferation; reduced metastasis risk	SFN increases paclitaxel-induced apoptosis in breast cancer cells by activating extrinsic and intrinsic signaling pathway members caspase-3, −8, and −9 and cytochrome C. SFN also reduces protein expression of apoptosis regulator Bcl-2 which otherwise inhibits the effectiveness of other breast cancer treatments. SFN causes a downregulation of the NF-κB pathway, thus inhibiting tumor proliferation and metastasis. The compound also suppresses the expression of phosphorylated AKT serine/threonine kinase. SFN is a novel therapeutic strategy for breast cancer treatment	Potential for therapy; implications for treatment; reduced metastasis risk
Li et al. (2020) [7], United States	HER2- enriched breast cancer	Maternal epigenetic regulation contributes to prevention of estrogen receptor-negative mammary cancer with broccoli sprout consumption	In vivo animal model, N = 96	Prevention of tumor formation, reduced tumor proliferation; reduced metastasis risk	Maternal dietary broccoli sprouts (BSps) inhibited and prevented mammary cancer formation and exhibited suppressive effects on mammary cancer. Maternal dietary BSp’s protective effects were associated with differentially expressed CSC-related and epigenetic-related genes and altered global histone acetylation. The intervention also enhanced DNA methylation and DNA hydroxymethylation levels; inhibited protein levels of Bmi1, HDACs, and DNMTs in ERα-negative breast cancer cell lines; and increased protein expression of tumor suppressor genes p16 and p53. Maternal dietary BSp unlocks phytochemicals that enhance estrogen receptor-negative mammary tumor prevention in mothers and offspring	Potential for therapy; reduced metastasis risk; safety implications in fetuses
Mielczarek et al. (2019) [8], Poland	HER2- enriched breast cancer	In the triple-negative breast cancer MDA-MB-231 cell line, sulforaphane enhances the intracellular accumulation and anticancer action of doxorubicin encapsulated in liposomes	In vitro cell culture, N = 8	Reduced tumor proliferation; increased cell death	SFN enhances cellular endocytotic internalization and enhances the effectiveness of breast cancer treatments. SFN increases the viability of healthy cells by 50%. SFN creates a synergistic effect when used alongside other breast cancer treatments such as radiation therapy and thus reduces the effective dose needed to achieve desirable outcomes. SFN also induces ROS and disturbs the mitochondrial potential that necessitates apoptosis. SFN is a highly effective anticancer medication	Potential for therapy; implications for treatment safety through reduction of effective treatment dose
Pogorzelska et al. (2023) [9], Poland	TNBC	Anticancer effect and safety of doxorubicin and nutraceutical sulforaphane liposomal formulation in triple-negative breast cancer (TNBC) animal model	In vivo animal model, N = 8	Reduced tumor proliferation; increased cell death; decreased migration of cancerous cells; ROS modulation	SFN has a synergistic interaction with chemotherapy and radiation therapy that enhances cytotoxicity by up to 1.8 times and directs the treatments into the nuclei of cancerous cells. However, SFN does not affect ROS level or Nrf2 localization. Liposomes containing SFN inhibit primary tumor growth in TNBC models by 14%–25% due to its synergic interaction with other cancer treatments, with the combined intervention achieving 2.5 times better outcomes. The combination of SFN with radiation therapy and chemotherapy shows systemic safety and protection of healthy breast tissue against treatment-induced toxicity, including counteracting urea levels by 25%. SFN increases cell death and the safety and effectiveness of radiation therapy and chemotherapy	Potential for therapy; implications for treatment; reduced metastasis risk; protection of normal cells or increased safety of treatment
Pogorzelska et al. (2024) [10], Poland	TNBC	Antitumor and antimetastatic effects of dietary sulforaphane in a triple-negative breast cancer models	In vivo animal model, N = 8	Increased cell death; reduced proliferation	SFN reduces tumor growth by 31% and lowers the proliferating potential of breast CSCs. SFN also reduces areas of necrosis and causes a modification of immune cell type infiltration, thus reducing metastasis of cancer cells. SFN inhibits cancer cell migration particularly in TNBC primary tumor by activating Nrf2 and enhancing the secretion of neurophils. SFN lowers the survival rate to 65% when administered at concentrations of 250 µM but does not cause hormetic action at lower doses. SFN suppresses the spread of CSCs released by TNBC primary tumors	Potential for therapy; implications for treatment; reduced metastasis risk; choice of safe and effective SFN dose or concentration
Sharma & Tollefsbol (2022) [11], United States	TNBC	Combinatorial epigenetic mechanisms of sulforaphane, genistein and sodium butyrate in breast cancer inhibition	In vitro cell culture, N = 2	Increased cell death; reduced proliferation	SFN was highly effective in reducing breast cancer cell viability. Combining SFN with dietary compounds genistein and sodium butyrate had synergistic effects in decreasing cancerous cellular viability at lower dosages compared to each compound’s single dosage in breast CSC lines, with the tri-combination having a greater effect than di-combinations. The combination limited cancer cell growth and increased apoptosis and necrosis in cancerous cells by arresting MDA-MB-231 and MCF-7 breast CSCs at the G2/M phase. SFN combined with genistein and sodium butyrate is highly effective in inhibiting breast cancer genesis by regulating epigenetic modifications which may enhance cancer therapy	Potential for therapy; implications for treatment; reduced metastasis risk
Tsaur et al. (2022) [12], Germany	Renal cell carcinoma	Concomitant use of sulforaphane enhances antitumor efficacy of sunitinib in renal cell carcinoma in vitro	In vitro cell culture, N = 7,860	Increased cell death; reduced tumor proliferation; reduced risk of metastasis; increased safety of treatment	SFN leads to antitumor properties in breast tissues without causing notable side effects. SFN prevents tumor growth and proliferation in cancer cases that are resistant to conventional treatments such as sunitinib by blocking clonogenic growth. SFN is an effective integrative component in treating metastasized cancer. SFN also reduces cancer cell viability	Potential for therapy; implications for treatment; reduced metastasis risk; SFN is safer due to its reduced risk of side effects
Yang et al. (2018) [13], China	TNBC	Sulforaphane induces autophagy by inhibition of HDAC6-mediated PTEN activation in triple negative breast cancer cells	In vitro cell culture, N = 3,000	Increased cell death; reduced proliferation	SFN inhibits cancerous cell growth and induces autophagy inhibition in MDA-MB-231, BT549, and MDA-MB-468 cells. SFN induces autophagy by downregulating HDAC6 expression and increasing acetylation modification and membrane translocation of PTEN. SFN and doxorubicin combination exhibits synergistic inhibition on TNBC cell growth with a greater inhibitory effect on MDA-MB-231 xenograft growth compared to a single treatment. SFN promotes the death of breast cancer cells and prevents their spread to other tissues	Potential for therapy; implications for treatment; reduced metastasis risk
Zhang et al. (2022) [14], Japan	TNBC	Sulforaphane suppresses metastasis of triple-negative breast cancer cells by targeting the RAF/MEK/ERK pathway	In vitro cell culture, N = 54	Increased cell death; reduced tumor proliferation; reduced risk of metastasis	R-SFN and S-SFN isomers of SFN inhibit TGF-β1-induced migration and invasion in breast cancer cells. SFN enhances the regulation of the actin cytoskeleton by suppressing the formation-associated proteins associated with the expression of actin stress fiber and inhibits TGF-β1-induced actin stress fiber formation thereby reducing the risk of breast tumor metastasis. SFN further inhibits breast tumor cell metastasis by directly binding to RAF family proteins ARAF, BRAF, and CRAF, as well as inhibiting MEK and ERK phosphorylation that could otherwise necessitate the stiffening and proliferation of preinvasive cancer cells. SFN is highly effective in inhibiting breast cancer cell proliferation and metastasis	Potential for therapy; implications for treatment; reduced metastasis risk

**Table 2:** Summary of evidence on sulforaphane and radiation therapy in breast cancer management. RCT: randomized controlled trial; SFN: sulforaphane; CSC: cancer stem-like cells; ALDH1A1: aldehyde dehydrogenase 1A1; ROS: reactive oxygen species; HDAC5: histone deacetylase 5; LSD1: lysine-specific demethylase 1; HER2: human epidermal growth factor receptor 2; Bcl-2: B-cell lymphoma-2; BSp: broccoli sprouts; Nrf2: nuclear factor erythroid 2-related factor 2; PTEN: phosphatase and tensin homolog; BSE-SFN: broccoli sprout extract containing sulforaphane; TGF-β: transforming growth factor beta; RIMF: radiation-induced muscle fibrosis

Author (year)	Article title	Methodology	Patient outcomes	Findings and conclusions	Implications
Kirkwood et al. (2017) [15]	Dose-response evaluation of broccoli sprout extract sulforaphane (BSE-SFN) in patients (Pts) with atypical/dysplastic nevi (A/DN)	Double-blinded RCT	Improved patient outcomes; lower side effects	There was no dose-limiting SFN toxicity. Plasma SFN at the first, second, and 28^th^ day of treatment showed increments of 120 ng/mL (-182–208), 206 (-356–420), and 655 (396–1,305) for 50 µmol, 100 µmol, and 200 µmol, respectively. Proteomics SFN at doses ranging from 50 µmol to 200 µmol showed a significant increase in tumor suppressor and a reduction in pro-inflammatory factors. Only one out of 17 patients reported nausea (two episodes) during the four weeks of treatment. Oral SFN is safe and effective at 50–200 µmol daily	Potential for therapy; implications for treatment safety through reduction of effective treatment dose
Lewinska et al. (2017) [16]	Sulforaphane-induced cell cycle arrest and senescence are accompanied by DNA hypomethylation and changes in microRNA profile in breast cancer cells	In vitro cell culture	Reduced tumor proliferation, increased cell death, improved patient outcomes, reduced side effects	SFN enhanced cell cycle arrest, elevated p21 and p27 levels, and enhanced cellular senescence when administered at concentrations of 5–10 µM. Apoptosis occurs when SFN is administered at concentrations of 20 µM, with the effects being accompanied by genotoxicity, nitro-oxidative stress, and diminished AKT signaling which eventually inhibits cell growth and diminishes their survival. SFN also stimulates energy stress based on reduced ATP and AMPK pool activation, while it also induces autophagy or cell death. SFN’s anticancer effects are mediated by global DNA hypomethylation as well as diminished N6-methyladenosine (m6A) RNA methylation pools. The phenomenon is also caused by reduced DNMT1 and DNMT3B DNA methyltransferase levels. SFN is an epigenetic modulator in breast tumors that results in cell cycle arrest and senescence which could enhance breast cancer treatment in the form of epigenome-focused anticancer therapy	Potential for therapy; implications for treatment safety through reduction of effective treatment dose
Tahata et al. (2018) [17]	Evaluation of biodistribution of sulforaphane after administration of oral broccoli sprout extract in melanoma patients with multiple atypical nevi	RCT	Improved patient outcomes; lower side effects	Plasma SFN levels pooled for the first, second, and 28^th^ day of treatment revealed post-administration increases of 120 ng/mL, 206 ng/mL, and 655 ng/mL for 50 µmol, 100 µmol, and 200 µmol, respectively. Plasma pro-inflammatory cytokine levels dropped consistently from the first to 28^th^ day while tumor suppressor decorin increased steadily from day one to 28. Breast cancer patients can easily tolerate oral BSE-SFN intervention at daily doses that do not exceed 200 µmol and investigations on the efficacy and safety of higher doses are necessary to optimize patient outcomes	Potential for therapy; implications for treatment safety through reduction of effective treatment dose
Wang et al. (2022) [18]	Sulforaphane regulates Nrf2-mediated antioxidant activity and downregulates TGF-β1/Smad pathways to prevent radiation-induced muscle fibrosis	In vivo animal model	Improved patient outcomes; lower side effects; reduced cell damage due to radiation exposure	Patients assigned to the radiation therapy group exhibited skeletal muscle fiber atrophy, higher inflammatory cell infiltration, and a significantly higher number of collagen fibers compared to patients who received a combination of radiation therapy and SFN. The combination group also had greater oxidative stress-related indexes and enhanced TGF-β/Smad pathway activation. The introduction of SFN restored all the above indices significantly and induced the upregulation of Nrf2 and activation of AKT. SFN inhibited GSK-3β and Fyn accumulation. Adding SFN to radiation therapy enhances treatment outcomes by RIMF prevention	Potential for therapy; implications for treatment safety by reducing radiation-induced cellular and muscular damage
Wei et al. (2021) [19]	Sulforaphane-mediated Nrf2 activation prevents radiation-induced skin injury through inhibiting the oxidative-stress-activated DNA damage and NLRP3 inflammasome	In vivo animal model	Improved patient outcomes; lower side effects	Morphological changes in skin tissues and oxidative stress on mice (by impacting damage indicators ROS, 4-HNE, and 3-NT) were detected eight weeks after irradiation. Pathological changes declined sharply after subjects were treated with SFN (p < 0.05) while the expressions of Nrf2 were higher in the SFN group compared to the control group (p < 0.05). SFN can suppress oxidative stress by upregulating Nrf2 expression and function as well as inhibiting NLRP3 inflammasome activation and DNA damage to alleviate radiation-induced skin injury	Potential for therapy; implications for treatment safety through reduction of effective treatment dose that reduces radiation-associated damage
Zhang et al. (2019) [20]	Sulforaphane bioavailability and chemopreventive activity in men presenting for biopsy of the prostate gland: A randomized controlled trial	Double-blind RCT	Improved patient outcomes; lower side effects	Plasma SFN isothiocyanates and individual SFN metabolites were statistically higher in the SFN group vs. the placebo group. Two out of 98 patients reported diarrhea and headache as side effects, although the difference between the SFN group and placebo-controlled group showed no statistically significant differences. SFN supplementation correlates with changes in gene expression but does not affect most cancer biomarkers	Potential for therapy; implications for treatment safety by reducing side effects

Evidence from the 20 studies [1-20], including three double-blinded RCTs [15,17,20], six in vivo animal model studies [3,7,9,10,19,20], and 11 in vitro cell culture studies [1,2,4-6,8,11-14,16,18], was synthesized to identify common themes relevant to the topic. This evidence was broadly categorized into two themes, namely, the role of SFN in breast cancer cell biology and the role of SFN and radiation therapy in the treatment of breast cancer. Below is a summary of these themes and their sub-themes.

Role of sulforaphane in breast cancer biology

Evidence from 14 studies extensively covered SFN’s role in breast cancer cell biology [1-14]. Precisely, these studies revealed that SFN can influence breast cancer cell behavior by modulating oxidative stress. This section explores the biological mechanisms through which SFN affects breast cancer cell proliferation, apoptosis, and metastasis, providing a foundation for understanding its therapeutic potential.

Modulation of Reactive Oxygen Species or Reduction of Tumor Proliferation

One of the studies revealed that combining SFN and taxanes leads to a greater reduction in primary tumor volume and inhibits the formation of secondary tumors more effectively compared to a single treatment [1]. Another study found that SFN facilitates lysine-specific demethylase 1 (LSD1) ubiquitination/degradation in a histone deacetylase 5-dependent (HDAC5) manner which inhibits breast cancer progression [2]. Further, SFN inhibits cell proliferation and mammosphere formation of CSCs in TNBC by decreasing the expression of cancer-specific *CR1* and *CRIPTO-3/TDGF1P3* genes [3]. SFN suppresses the expression of stem cell markers Notch4, aldehyde dehydrogenase 1A1 (ALDH1A1), Nanog, and Wnt3 [3]. It creates a synergistic effect when used alongside other breast cancer treatments such as radiation therapy and thus reduces the effective dose needed to achieve desirable outcomes [8]. SFN also induces reactive oxygen species (ROS) and disturbs the mitochondrial potential that necessitates apoptosis [8]. However, these findings were challenged in an animal model study which found that SFN does not affect ROS level or nuclear erythroid 2-related factor 2 (Nrf2) localization [9].

Induction of Apoptosis

One of the studies revealed that SFN preferentially eliminates breast CSCs by suppressing nuclear factor kappa B (NF-κB) p65 subunit translocation, downregulating p52 proteins, and inhibiting downstream transcriptional activity [1]. Another study found a direct correlation between the effect of SFN on CSC death and HER2 levels, with a higher number of cell deaths being reported at higher concentrations of SFN [5]. Precisely, cell co-staining revealed that the percentage of apoptosis correlates with CSC apoptosis following different treatments, with greater outcomes being reported in patients treated with SFN compared to metformin. SFN also increases paclitaxel-induced apoptosis in breast CSCs by activating extrinsic/intrinsic signaling pathway caspase-3, −8, and −9 members, as well as cytochrome C [6]. Further, the compound reduces the expression of apoptosis regulator Bcl-2 protein which otherwise inhibits the effectiveness of other breast cancer treatments [6]. Combining SFN with metformin synergized particular molecules or cell-signaling pathways and showed their cooperative effects on cancer cells [5].

SFN inhibits cancerous breast CSC proliferation and induces MDA-MB-231, BT549, and MDA-MB-468 cell autophagy [13]. In this case, SFN induces autophagy by downregulating HDAC6 expression and increasing acetylation modification and membrane translocation of both tensin and phosphatase homologs (PTENs) [13]. SFN also stimulates energy stress based on reduced pools of ATP/AMPK activation, while it also induces autophagy or cell death. SFN’s anticancer effects are mediated by global DNA hypomethylation, diminished N6-methyladenosine RNA pool methylation, and reduced DNA methyltransferases DNMT1 and DNMT3B levels [17]. SFN functions as an epigenetic modulator in breast tumors that causes cell cycle arrest and senescence which could enhance breast cancer treatment in the form of epigenome-focused anticancer therapy [17]. SFN shows high effectiveness in reducing breast cancer cell viability particularly when combined with dietary compounds genistein and sodium butyrate [11,12]. This combination creates synergistic effects by reducing cancerous cellular viability at minimal doses compared to each compound’s single dosage in breast cancer cell lines, with the tri-combination having a greater effect than di-combinations [11]. The combination further limits cancer cell growth and increases apoptosis and necrosis in cancerous cells by arresting MDA-MB-231 and MCF-7 breast CSCs at the G2/M phase. Combining SFN and doxorubicin exhibits a synergistic effect in the inhibition of TNBC cell growth and leads to a greater inhibitory effect on MDA-MB-231 xenograft growth compared to a single treatment [13].

Inhibition of Metastasis

Studies have shown that SFN can reverse aldehyde dehydrogenase-positive cell enrichment which is typically induced by taxane [1]. This process eventually reduces the volume of both primary and secondary mammospheres that are formed from breast CSCs [1]. SFN further leads to a downregulation of the NF-κB signaling pathway, thus inhibiting tumor proliferation and metastasis. The intervention also reduces phosphorylated AKT serine/threonine kinase expression [6]. Another study found that maternal dietary broccoli sprouts (BSps) inhibited and prevented mammary cancer formation and exhibited suppressive effects on mammary cancer [7]. The protective effects of maternal dietary BSps were linked with differentially expressed tumor-related genes and epigenetic-related genes and altered global histone acetylation. The intervention also enhanced DNA methylation and DNA hydroxymethylation levels; inhibited protein levels of Bmi1, HDACs, and DNMTs in ERα-negative breast cancer cell lines; and increased protein expression of tumor suppressor genes *p16* and *p53* [7]. Further, an animal model study found that liposomes containing SFN inhibit primary tumor growth in TNBC models by 14%-25% due to its synergic interaction with other cancer treatments [9].

Another study found that SFN reduces tumor growth by 31% and lowers the proliferating potential of cancer tumors, reduces areas of necrosis, and modifies immune cell type infiltration which reduces metastasis of breast tumors [10]. The compound further inhibits cancer cell migration particularly in TNBC primary tumor and its environment by activating nuclear factor erythroid 2-related factor 2 (Nrf2) and enhancing the secretion of neutrophils [10]. R-SFN and S-SFN, which are isomers of SFN, suppress transforming growth factor beta 1 (TGF-β1)-induced migration and resultant invasion in breast CSCs. SFN enhances the regulation of the actin cytoskeleton by suppressing the expression of actin stress fiber formation-associated proteins and inhibiting TGF-β1-induced actin stress fiber formation, thereby reducing the risk of breast tumor metastasis [14]. SFN further inhibits breast tumor cell metastasis by directly binding to ARAF, BRAF, and CRAF proteins as well as inhibiting MEK and ERK phosphorylation that could otherwise necessitate the stiffening and proliferation of preinvasive cancer cells [14]. Furthermore, SFN and LSD1 inhibitor combination treatment revealed superior MDA-MB-231 xenograft tumor growth inhibition by enhancing HDAC5-LSD1 axis inhibition and blocking breast cancer growth [2].

Sulforaphane and radiation therapy in breast cancer

This section reviews the radioprotective properties of SFN, its mechanisms of action, and potential benefits for radiation therapy outcomes. Studies highlighting the effects of SFN on normal breast tissues and its potential to minimize radiation damage are summarized. Below is a summary of this evidence.

Reduced Tissue Damage or Protection of Normal Tissues

Studies show that combining SFN with a potent LSD1 inhibitor creates synergy by amplifying tumor growth inhibition with no impact on normal breast epithelial cells [2]. SFN inhibition of HDAC5/LSD1 expression shows similar effects on expression of target genes which further enhances the antitumor efficacy of SFN [2]. SFN reverses the resistance of carcinoma cells to cisplatin caused by DNA damage and enhances the accumulation of intracellular cisplatin. Treating breast cancer patients with the compound further enhances the expression of miR-30a-3p in cisplatin-resistant cells by enhancing the sensitivity of A2780/CP70 and IGROV1-R10 cells to cisplatin treatment [4].

Combining Sulforaphane With Radiotherapy

The combination of SFN with radiation therapy and chemotherapy exhibits systemic safety and protection of healthy cells against treatment-induced toxicity, including counteracting urea levels by 25% [9]. Similarly, combining SFM with radiotherapy achieves 2.5 times better outcomes in reducing the proliferation of breast tumors [9]. SFN has a synergistic interaction with chemotherapy and radiation therapy that enhances cytotoxicity by up to 1.8 times and directs the treatments into the nuclei of cancerous cells [9]. In one empirical study, patients assigned to the radiation therapy group showed more obvious signs of skeletal muscle fiber atrophy, higher inflammatory cell infiltration, and a significantly higher number of collagen fibers compared to patients who received a combination of radiation therapy and SFN [18]. The combination group also had significantly increased expression of oxidative stress-related indexes and activated TGF-β/Smad pathways. The introduction of SFN restored all the above indices, induced upregulation of Nrf2 and activation of AKT, and inhibited GSK-3β and Fyn accumulation [18]. Studies have also shown morphological changes in skin tissues and oxidative stress in mice (by impacting damage indicators ROS, 4-HNE, and 3-NT) eight weeks after irradiation. However, pathological changes declined sharply after subjects were treated with SFN (p < 0.05), while the expressions of Nrf2 were higher in the SFN group compared to the control group (p < 0.05) [19].

Safe and Effective Concentrations of Sulforaphane

Many studies have not found any dose-limiting SFN toxicity. Plasma SFN at days one, two, and 28 caused increments of 120 ng/mL (-182-208), 206 (-356-420), and 655 (396-1,305) for 50 µmol, 100 µmol, and 200 µmol, respectively. Proteomics SFN at doses ranging from 50 µmol to 200 µmol showed a significant increase in tumor suppression and a reduction in pro-inflammatory markers [15]. SFN promoted cell cycle arrest, elevated the levels of p21 and p27, and enhanced cellular senescence when administered at concentrations of 5-10 µM. Apoptosis occurs when SFN is administered at concentrations of 20 µM, with the effects accompanied by genotoxicity, nitro-oxidative stress, and diminished AKT signaling, which, eventually, inhibits cell growth and diminishes their survival [17]. However, another study found that SFN lowers cancer cell survival rate to 65% when administered at concentrations of 250 µM but does not cause hormetic action in lower doses [10]. SFN also prevents tumor growth and proliferation in cancer cases that are resistant to conventional treatments such as sunitinib by blocking clonogenic growth [12]. SFN is an effective integrative component in treating metastasized cancer [12]. SFN also enhances cellular endocytotic internalization and enhances the effectiveness of breast cancer treatments. SFN increases the viability of healthy cells by 50% [8].

SFN is highly effective with minimal adverse events. In one study, only one out of 17 patients reported nausea (two episodes) during the four weeks of treatment [15]. Patients in the SFN group had statistically higher plasma SFN isothiocyanates and individual SFN metabolites compared to the placebo group. Another study found that only two out of 98 patients reported diarrhea and headache as side effects [20]. However, these side effects were mild and no statistically significant differences were noted in the SFN group compared to the placebo/control group.

Discussion

**Figure 2:**
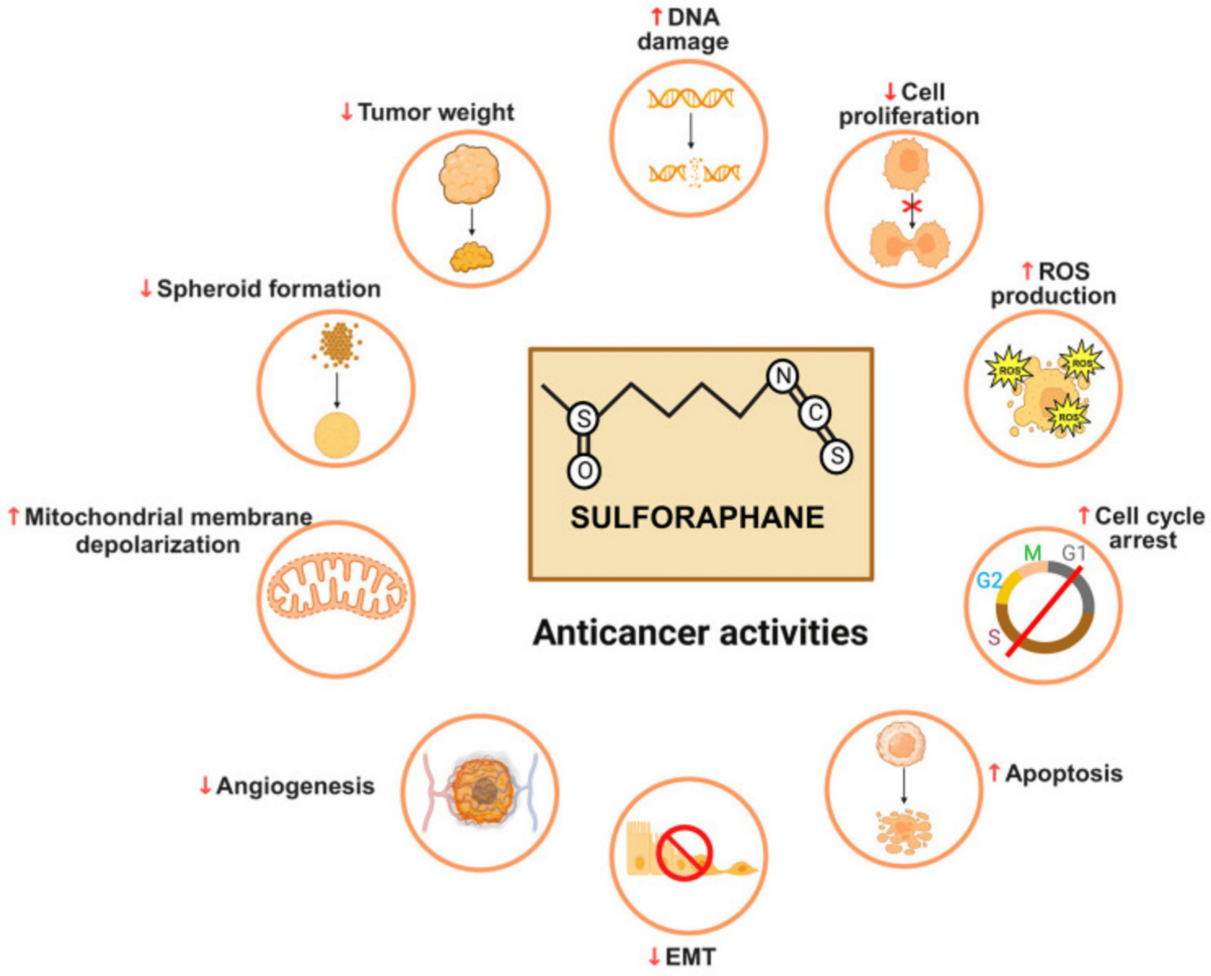
A summary of sulforaphane’s anticancer activities. Image reproduced from Sailo et al. [25]. The image has been reproduced from an open-access article distributed under the terms and conditions of the Creative Commons Attribution (CC BY) license.

Many of the studies confirm that SFN can prevent and treat breast cancer by targeting cell biology and creating a synergistic effect on other treatments [10,15,17,21-24]. Most of the selected studies confirmed that the compound exhibits a greater reduction in breast tumor volume by modulating ROS and inhibiting the expression of genes that necessitate the proliferation of breast cancer [1-3,8]. However, only one study challenged the effectiveness of SFN in modulating ROS and inhibiting tumor proliferation due to a lack of statistical significance in its findings [9]. Other studies also confirmed that SFN induces apoptosis by preferentially eliminating breast cancer CSCs, inhibiting downward transcription activity, downregulating proteins that necessitate tumor growth, reducing levels of DNA methyltransferases, causing cell cycle arrest and senescence, inhibiting the growth of xenografts, and resulting in breast cancer death [1,5,6,11-13,17]. Figure 2 summarizes the various mechanisms through which SFN inhibits cancer development and proliferation.

**Figure 3:**
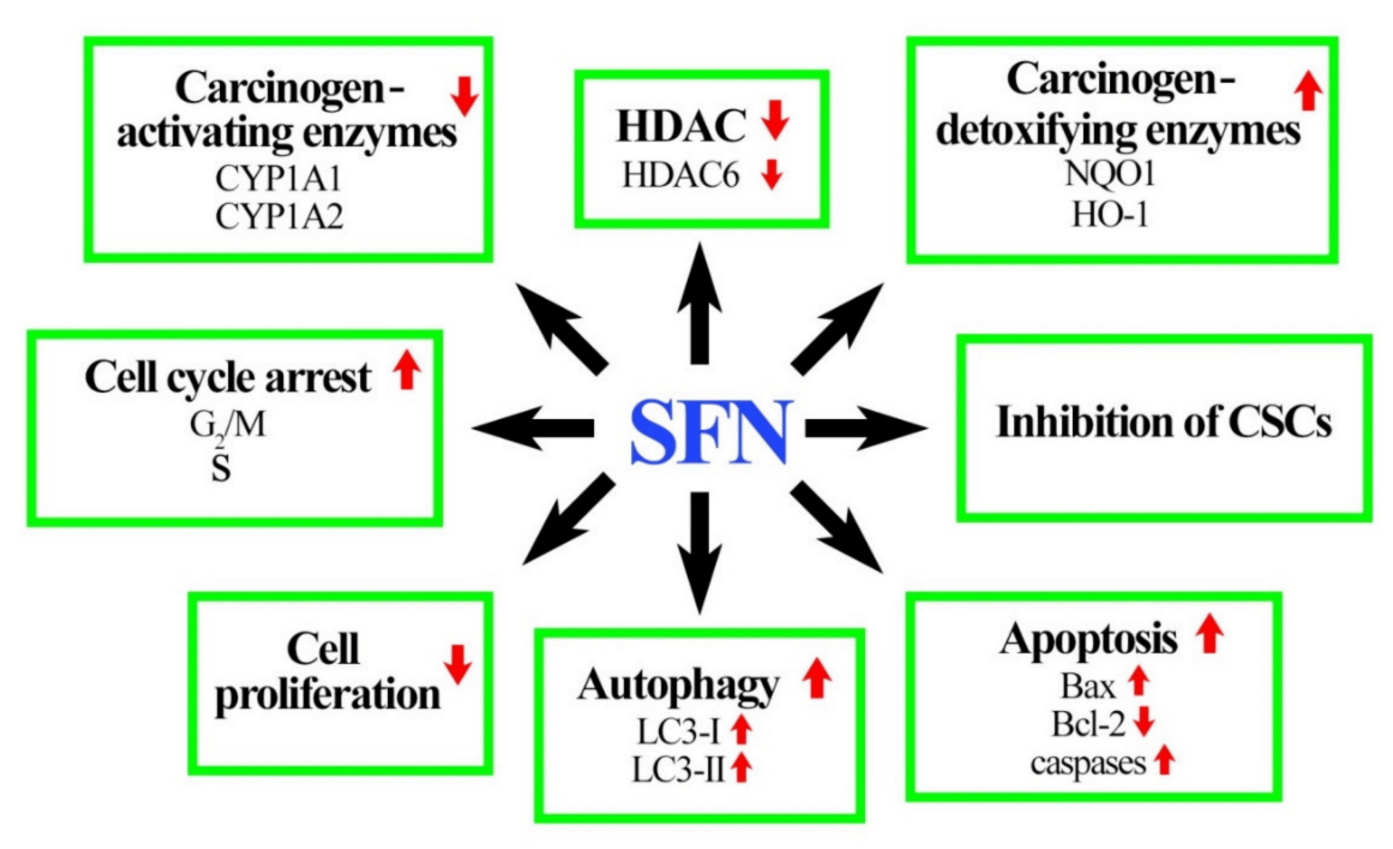
Role of sulforaphane (SFN) in cancer treatment. Image reproduced from Kuran et al. [13]. The image has been reproduced from an open-access article distributed under the terms and conditions of the Creative Commons Attribution (CC BY) license.

Figure 3 further illustrates how SFN contributes to cancer treatment. The image on the left shows how HDAC6 inhibits the autophagy of breast cancer cells with damaged DNA. However, the introduction of SFN causes a downregulation of HDAC6 expression which then increases acetylation modification and membrane translocation of phosphatase to activate cancer cell death.

Many of the selected studies unanimously supported the safety of SFN in breast cancer management due to its beneficial effects on the prevention and treatment of the chronic condition. Some studies showed that the compound inhibits tumor growth with no significant impact on normal breast cells [2,4]. Others showed that SFN causes a synergistic effect, increases the effectiveness of radiotherapy by 1.8-2.5 times, and restores the function of cells that were previously damaged by irradiation [9,18]. SFN’s safety is validated by the lack of or minimal side effects when the compound is administered in concentrations ranging from 5 µM to 200 µM [8,10,12,15,17]. The administration of SFN alongside other interventions seems to yield better outcomes compared to the administration of the compound as a single treatment [2,4,9,11,18].

There is overwhelming evidence from all 20 studies selected for this review that SFN is effective and safe in breast cancer treatment when used either solely or in combination with conventional cancer treatments such as radiotherapy. However, most of the evidence has been sourced from cell culture studies and animal models. The efficacy and safety that SFN has demonstrated shows a need for further investigations to gather empirical evidence from in vitro human studies to validate its effectiveness in treating and preventing breast cancer. Interestingly, the few empirical studies selected for this review demonstrated that the intervention is safe for humans and has the potential to enhance the management of prostate cancer [20]. Scholars agree that further investigations on the effectiveness and safety of this compound could necessitate its approval in breast cancer either as a single treatment or in combination with other chemotherapeutic, radiotherapeutic, or integrative therapeutic agents [21,22,26-29].

This study had many limitations. First, the accuracy of the findings presented in this study may be compromised as no empirical evidence was collected to verify the findings. The review study design further limits the validity and reliability of its findings due to the heterogeneity of findings particularly due to the coverage of patients with different breast cancer subtypes in the studies selected for the review. The study also failed to establish the causal effect between SFN and breast cancer treatment outcomes and radiotherapy safety. As such, there is a need to perform additional empirical studies on this topic.

## Conclusions

Breast cancer is a debilitating chronic condition with a high incidence and mortality rate and one that has proven difficult to treat. However, the reviewed studies suggested that SFN could be a potential treatment that could enhance breast cancer treatment outcomes by causing cell death and improving the safety and effectiveness of radiation therapy and chemotherapy. SFN plays a critical role in necessitating damage to breast cancer CSCs, inhibiting the function of proteins and genes that necessitate breast cancer proliferation, and regulating the spread of cells released by TNBC primary tumors. SFN can suppress oxidative stress by causing an upregulation of Nrf2 expression and function as well as inhibiting NLRP3 inflammasome activation and DNA damage to alleviate radiation-induced skin injury. SFN combined with chemotherapy, radiotherapy, and other anticancer dietary interventions is highly effective in inhibiting breast cancer genesis by regulating epigenetic modifications, likely enhancing cancer therapy. As such, adding SFN to radiation therapy enhances treatment outcomes by preventing radiation-induced muscle fibrosis. Further investigations on the effectiveness and safety of this compound could lead to its approval in breast cancer either as a single treatment or part of a combination treatment regimen.
